# Quality of *Physalis peruviana* fruits coated with pectin and pectin reinforced with nanocellulose from *P. peruviana* calyces

**DOI:** 10.1016/j.heliyon.2021.e07988

**Published:** 2021-09-14

**Authors:** Liceth Carolina Cárdenas-Barboza, Andrey Camilo Paredes-Córdoba, Liliana Serna-Cock, Marcelo Guancha-Chalapud, Cristian Torres-León

**Affiliations:** a School of Engineering and Administration. Universidad Nacional de Colombia, Street 32 Chapinero, 763533, Palmira, Valle del Cauca, Colombia; b National Center for Technical Assistance to Industry (ASTIN), Servicio Nacional de Aprendizaje – SENA, 760004, Cali, Valle del Cauca, Colombia; c Research Center and Ethnobiological Garden, Universidad Autónoma de Coahuila, 27480, Viesca, Coahuila, Mexico

**Keywords:** *Physalis peruviana* calyces, Agroindustrial byproducts, Nanocellulose, Edible coating, Fruits

## Abstract

*Physalis peruviana* is marketed without its calyx, which generates byproducts and a decrease in the shelf life of these fruits. The aim of this study was to evaluate the effect of edible pectin-coatings reinforced with nanocellulose from calyx on the physical-chemical and physiological parameters of *P. peruviana* fruits during refrigerated storage (5 °C) for ten days. The nanocellulose extraction was carried out using a combined extraction method (chemical procedures and ultrasound radiation). The characterization of the fibers showed that the maximum degradation temperatures ranged between 300 and 311 °C. The SEM analysis revealed the presence of fibers after the chemical treatment. The removal of lignin and hemicellulose was validated using Fourier Transform Infra Red (FTIR) spectroscopy. The results showed that the fruits treated with pectin and pectin reinforced with nanocellulose at 0.5 % (w/w) had an adequate visual appearance and showed a minor color change (ΔE of 19.04 and 21.04, respectively) and the highest retention of L∗ during storage. Although the addition of nanocellulose at 0.5% presented the lowest respiratory rate (29.60 mgCO_2_/kg h), the treatment with pectin offered the least weight loss and showed the highest firmness retention at the end of storage. Thus, the edible pectin-coating may be useful for improving the postharvest quality and storage life of fresh *P. peruviana* fruit. Nanocellulose from *P. peruviana* calyces can be used under the concept of a circular economy; although, its use as a reinforcement of pectin showed some limitations.

## 1 Introduction

*Physalis peruviana* Linnaeus is a fruit belonging to the *Solanaceae* family and genus *Physalis.* This plant is native to the South American Andes and produces orange-yellow fruit with a juicy berry commonly known as cape gooseberry or “uchuva” (Fischer et al., 2014; Morton, 1987). The fruit is covered by a fibrous structure formed by five sepals called the calyx, which is a casing that naturally protects the fruit during its development and maturity, protecting it against pathogens and external climatic conditions (Giraldo et al., 2017; Luchese et al., 2015). The calyx of *P. peruviana* is an inedible byproduct and represents 5% of the weight of fresh fruit (Ballesteros-Vivas et al., 2019). Colombia is the largest producer in the world, followed by South Africa (Bazana et al., 2019), countries that export fruit to other countries such as the Netherlands, the United Kingdom, Germany, Belgium, and the United States (Marchioretto et al., 2020; M.-L. Olivares-Tenorio et al., 2016). In Colombia, the fruit of *P. peruviana* has a fifth place in export fruits (Minagricultura, 2019). Currently, the market requires the commercialization of fruit without calyx, which reduces the shelf life of the fruits.

According to scientific literature, under storage conditions at 12 °C and 80% RH, cape gooseberry with calyx has a shelf life of 24 days, and fruits without calyx only retain their quality postharvest for 11 days (M.-L. Olivares-Tenorio et al., 2017). In order to combat all the different factors that cause damage to fruits and to preserve their quality, there have been used multiple methods. Among the technologies currently investigated, the use of edible coatings stands out (Ruiz-Martínez et al., 2020; Torres-León et al., 2018a, Torres-León et al., 2018b). Edible coatings are prepared from naturally occurring renewable polymers such as starches, dextrin, cellulose, alginate, chitosan, and pectin, which present desirable characteristics as biodegradability, good oxygen, and vapor barriers (without creating severe anaerobic conditions for the fruit) (Yousuf et al., 2018). Pectin has been used for the formulation of edible coatings; this polymer is biodegradable, non-toxic, biocompatible, soluble in water, and inexpensive (Nisar et al., 2019). To improve the technological characteristics of edible coatings, the incorporation of nanoparticles has been extensively investigated in recent years (Chávez-Magdaleno et al., 2018; Jafari et al., 2018; Md Nor and Ding, 2020; Szymańska-Chargot et al., 2019). Nanocellulose has higher crystallinity, specific surface area, surface chemical reactivity, mechanical strength, thermostability, and low density than cellulose (Chen et al., 2020; Trache et al., 2020). Therefore, studies on the incorporation of nanocellulose in polymeric matrices have been carried out in agricultural products such as spinach leaves (Pacaphol et al., 2019), pears (Deng et al., 2018; Jung et al., 2020), saffron (Jafari et al., 2018), grapes (Silva-Vera et al., 2018), bananas (Deng et al., 2017a, Deng et al., 2017b), and minimally processed apples (Zhai et al., 2020). The strengthening of formulations with nanocellulose improves the barrier properties and reduces the weight loss of Tahiti sour lime fruits (Laureth et al., 2018). Nanocellulose reinforced pectin formulations (5%, w/w) have better mechanical properties and lower permeability to water vapor (Chaichi et al., 2017). In this sense, the Calix of the fruit that is generated as a byproduct can be valued as a source of nano cellulose. The use of food byproducts as a source of biocompatible materials for the development of packaging could generate economic gains for the industry and would reduce the environmental implications that generate mismanagement of waste (Torres-León et al., 2018a, Torres-León et al., 2018b). Although, some studies report the use of coatings in the postharvest of *P. peruviana* (Carvalho et al., 2015; González-Locarno et al., 2020; Licodiedoff et al., 2016). There are no reports of the use of nanocellulose extracted from the calix (by-product) of *P. peruviana* as reinforcement of an edible pectin-coating. This is innovative and can directly impact the Sustainable Development Goals and the concept of a circular economy (Torres-León et al., 2021).

Therefore, the aim of this study was to evaluate the application of edible pectin-coatings reinforced with nanocellulose (obtained from the calyx of *P. peruviana*) on the physical-chemical and physiological quality of *P. peruviana* fruits during refrigerated storage. The nanocellulose was also characterized.

## 2 Materials and methods

### 2.1 Materials

Sodium hypochlorite (NaClO), Hydrochloric acid (HCl), sodium hydroxide (NaOH), acetic acid (CH_3_COOH), sulphuric acid (H_2_SO_4_), potassium hydroxide (KOH), Barium chloride (BaCl_2_) and Sodium chlorite (NaClO_2_) were purchased from Sigma-Aldrich. Commercial citrus pectin was purchased from San Jorge Ltda. (Palmira, Colombia).

### 2.2 Plant material

The *P. peruviana* fruit at the mature stage 3, according to the Colombian standard color classification chart (Icontec, 1999), were obtained from a commercial supplier in Palmira, Valle del Cauca, Colombia. The fruit was visually selected for uniformity in size, color, absence of blemishes, and fungal infection. The calyx had an approximate length of 5 cm; moisture content (40.08 ± 2.6%) was determined by air oven (Binder, Germany) method (24 h in air oven at 50 °C to constant weight).

### 2.3 Production of nanocellulose

Nanocellulose was obtained from *P. peruviana* calyces according to the methodology reported by (Zhang et al., 2014). Briefly, the calyces were chopped and disinfected with sodium hypochlorite at 200 mg/L for 15 min. Afterward, these were dried in an oven at 50 °C (Binder, Germany) to constant weight (NAT-C). Dried samples were ground (IKA A11, Mexico). The fat and pectin from the ground calyx were removed with heat treatment at 70 °C in HCl (0.01 N) for 2 h (NAT-C-EE). Subsequently, the pH was adjusted to 9.5 with NaOH (1 M). The samples were vacuum filtered and washed with distilled water until a neutral pH. The fibers were dried at 50 °C in a forced convection oven (Binder, Germany). The fibers were dignified with NaOH (4% p/v) for 4 h at 90 °C. Subsequently, double bleaching (B–C) was performed with NaClO_2_ (1.7% w/v) and acetate buffer (27 g NaOH and 75 ml of CH_3_COOH per liter), in a 1:1 ratio at 80 °C for 6 h. The fibers (B–C) were washed, vacuum filtered (with distilled water until neutral pH), and dried in a convective oven (Binder, Germany) at 50 °C for 24 h.

Finally, the B–C was mixed with H_2_SO_4_ (6.5 M) with agitation (400 rpm) for 24 h at 50 °C. The ratio of cellulose to acid was 1:25 (g/ml). Hydrolyzed samples (H–C) were neutralized (10% NaOH) and vacuum filtered (Whatman Filter Paper No. 41). A suspension of nanocellulose at (1% w/v) was mixed in an ultra-turrax (MicroDisTec™ homogenizer 1000, Switzerland) at 10.000 rpm for 15 min. After this, the dispersions were homogenized in an ultrasonic homogenizer (BRANSON, USA) at a frequency of 20 kHz (400 W) for 30 min (US–C). The nanofiber gels were dried in a convective oven at 50 °C.

### 2.4 Characterization of nanocellulose

#### 2.4.1 Scanning electron microscopy (SEM)

SEM (JCM 50000, Japan) was used for study of the morphology of the surfaces of NAT-C and B–C. The samples were coated with gold using high vacuum prior to the experiment.

#### 2.4.2 Fourier transform infrared spectroscopy (FTIR) spectroscopic analysis

FTIR was performed on NAT-C, B–C, H–C, and US-C to confirm the removal of lignin and hemicellulose. The spectra were collected at a wavelength ranging from 400-4000 cm^−1^. To record the spectra a spectroscope (Shimadzu, Japan) was used.

#### 2.4.3 Thermogravimetric analysis (TGA)

The thermal properties of samples (NAT-C, B–C, H–C, and US-C) was obtained in a computer DSC/TGA 2STAR system (Mettler Toledo, USA) with heating rate of 10 °*C min*^−1^ (The nitrogen flow rate was 20 mL min^−1^, the test temperature range was 30–600 °C).

### 2.5 Preparation of edible coatings

The coatings were prepared as described by Chaichi et al. (2017), with some modifications. Briefly, 6 g of pectin were dissolved in 120 ml of distilled water at 70 °C (Corning, USA). Next, glycerin was added at 0.3 w/w of pectin. The pH value of the solution was adjusted to 4.5 with 0.1 N NaOH (Orion Star A111, Thermo Scientific, USA). After this, different concentrations (0.0, 0.5, 2.0 and 5.0 % w/w) of nanocellulose were added. The dispersions were homogenized in an ultrasonic homogenizer (MicroDisTec™ Homogenizer 1000, Switzerland) at 10.000 rpm for 15 min. Samples were labeled according to the concentration of cellulose nanofibers, as follows: P (0 %), P + CN0.5%, P + CN2%, and P + CN5%.

### 2.6 Fruit conditioning and coating application

*P. peruviana* fruits (10 kg) without calyces were washed with a solution of sodium hypochlorite (200 ppm) for 15 min and air-dried at ambient temperature (30 °C). The fruits were divided into five groups. These were immersed in each concentration of nanocellulose coating (0.0, 0.5, 2.0, and 5.0 % w/w) for 1 min, and the coating solution was applied uniformly on the whole surface, while control fruit was dipped in purified water. The fruits were dried under airflow (25 °C) until solidification and were packed in expanded polystyrene (EPS) trays (130 mm × 130 mm × 13mm) wrapped with polyvinyl chloride (PVC) stretch film and stored at 5 °C and 60% RH for ten days. The fruit quality parameters were measured at 0, 1, 3, 5, 8, and 10 days of storage.

### 2.7 Physicochemical analyses

#### 2.7.1 Appearance

Physical changes of the fruits were evaluated on days 0, 1, 3, 5, 8, and 10: color change (green, orange, yellow), firmness, and appearance of the epidermis. A subjective scale was developed to evaluate the firmness of the fruits, classifying as firm those fruits that did not present deformation before the pressure on the surface of the fruit with the thumb and index fingers, and cataloging as loss of firmness those fruits that showed some deformation before the same subjective test. Photos were taken for each time interval to demonstrate the overall appearance.

#### 2.7.2 Weight loss

Fruit weight loss was evaluated using a digital balance (Denver Instrument APX-323, USA). Fruits with three replicates (n = 3) in each treatment were individually weighed at the beginning of storage and during each storage time. Weight loss was determined and expressed as a percentage (Lo'ay & Taher, 2018).

#### 2.7.3 Fruit firmness

The firmness (N) was measured using a penetrometer (PCE-FM 200 Instruments, Spain). The firmness of each fruit was measured at two opposite points of the equatorial area by using a 6 mm probe.

#### 2.7.4 Determination of color

The peel color of the fruits (n = 5) was measured using a Minolta Chroma Meter CR-400 (Konica Minolta, Japan) to assess a∗ (greenness was indicated by negative readings, while positive values were represented redness), b∗ (negative readings were indicative of blueness, while higher positive readings represented yellowness) and L∗ (luminosity). Subsequently, the color change (ΔE) with respect to zero time was calculated by applying Eq. (1) (Geraldi et al., 2020).

(1) $$\Delta \text{E}=\sqrt{{\left(\Delta a*\right)}^{2}+{\left(\Delta b*\right)}^{2}+{\left(\Delta L*\right)}^{2}}\;$$

### 2.8 Potential of hydrogen (pH), total soluble solids (TSS), titratable acidity (TA), and (TSS/TA) ratio

pH, TSS, and TA were measured in the extract of the fruits (20 g of pulp were macerated and filtered with a fine cloth) in triplicate for each batch during the time interval. The pH was measured using a digital potentiometer (Orion Star A111, Thermo Scientific, USA). TSS was assessed using a digital handheld refractometer (Reichert, Germany), and results were expressed as °Bx. TA content was measured by titrating the samples with NaOH solution at 0.1 M in a pH = 8.1 ± 0.2. The results were expressed as a percentage of citric acid. TSS/TA ratio was calculated as a percentage between the TSS and TA.

### 2.9 Evaluation of the respiration rate of the fruits

The respiration rate was measured by the titration method. The system has an air pump, CO_2_ traps, and three hermetically sealed vessels (breathing chambers) connected by hoses. Three fruits were used per container. The CO_2_ produced in the respiration of the fruits is collected in traps containing 50 ml of 0.1N NaOH. CO_2_ from the air is removed in a trap containing 50 ml of KOH 2N. Subsequently, the air is transported to the breathing chambers for 30 min. Finally, traps are removed from system. 20 ml of each sample was titrated with 15 ml of 10% BaCl_2_ (w/v) and 0.1 N HCl until reaching a pH of 8.1 ± 0.2. The respiratory rate was calculated using Eqs. (2) and (3).

(2) $$RR\left(\frac{mgCO_{2}}{kg*h}\right)=\frac{\left(V_{b}-V_{s}\right)*N_{HCl}*22*f}{\text{W∗t}}$$

(3) $$f=\frac{V_{NaOH\;}}{V_{sample}}$$

where RR is the fruit respiratory rate (mgCO_2_/kg h), Vb is the volume of HCl used in blank titration (ml), Vs is the volume of HCl used in the titration of the sample (ml), NHCL is the HCl normality, 22 is the CO_2_ equivalent weight (g-meq), f is the factor that relates the volume of NaOH used in the experimentation and the volume taken for the sample, W is the sample weight (kg), and t is the time of airflow in the system (h).

### 2.10 Statistical analysis

A completely randomized unifactorial design with five levels and successive repetitions six times (0, 1, 3, 5, 8, and 10 days of storage) was used. Factor: type of coating. Levels: control, P, P + CN0.5%, P + CN2% and P + CN5%. Five replications for each treatment were performed. The other data were subjected to analysis of variance (ANOVA) (p < 0.05), and the mean comparisons were performed using Duncan's multiple comparisons. All statistical determinations were performed using IBM SPSS Statistics software version 21.

## 3 Results and discussion

### 3.1 Characterization of nanocellulose

#### 3.1.1 Scanning electron microscopy (SEM)

**Figure 1:**
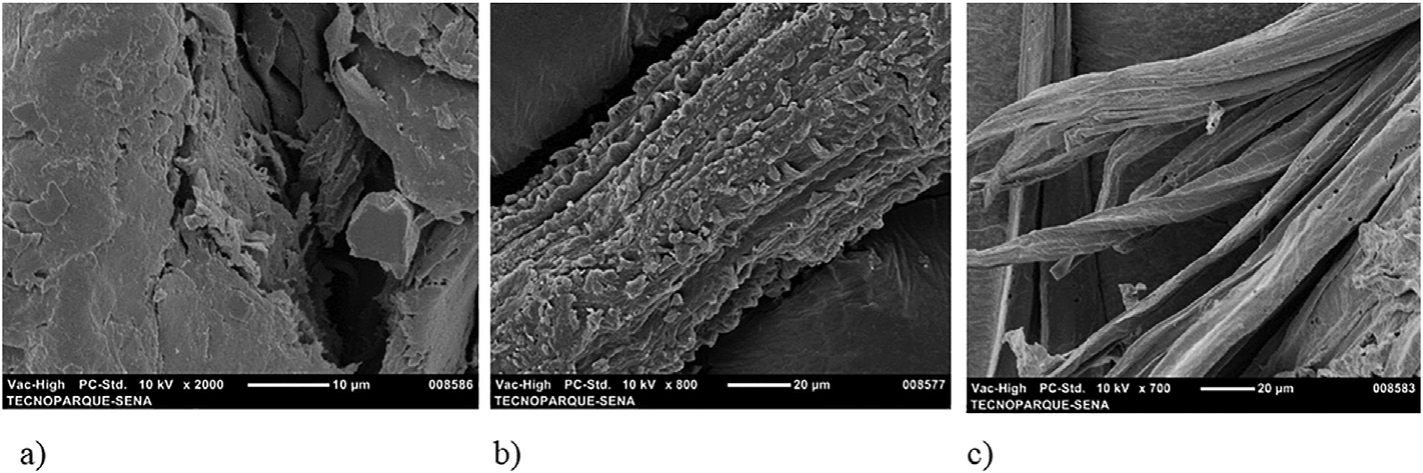
SEM micrographs of *P. peruviana* fibers (a): untreated native fibers (NAT-C). b: native fibers after pectin and fats extraction process (NAT-C-EE). c: bleached fibers (B–C).

Figure 1 shows the SEM micrographs of NAT-C, NAT-C-EE, and B–C. According to Figure 1 (a and b), a defined surface morphology was not observed. These results are in accordance with what was reported by Guancha-Chalapud et al. (2020) and Julie Chandra et al. (2016). The lignocellulosic biomass does not present a homogeneity defined by the presence of lignin, hemicellulose, and pectins. In Figure 1c, fibers between 10 and 21 μm were observed. This figure shows the elimination of the main components (lignin, hemicellulose, pectin, and fats) during the pretreatment.

#### 3.1.2 FTIR analysis

**Figure 2:**
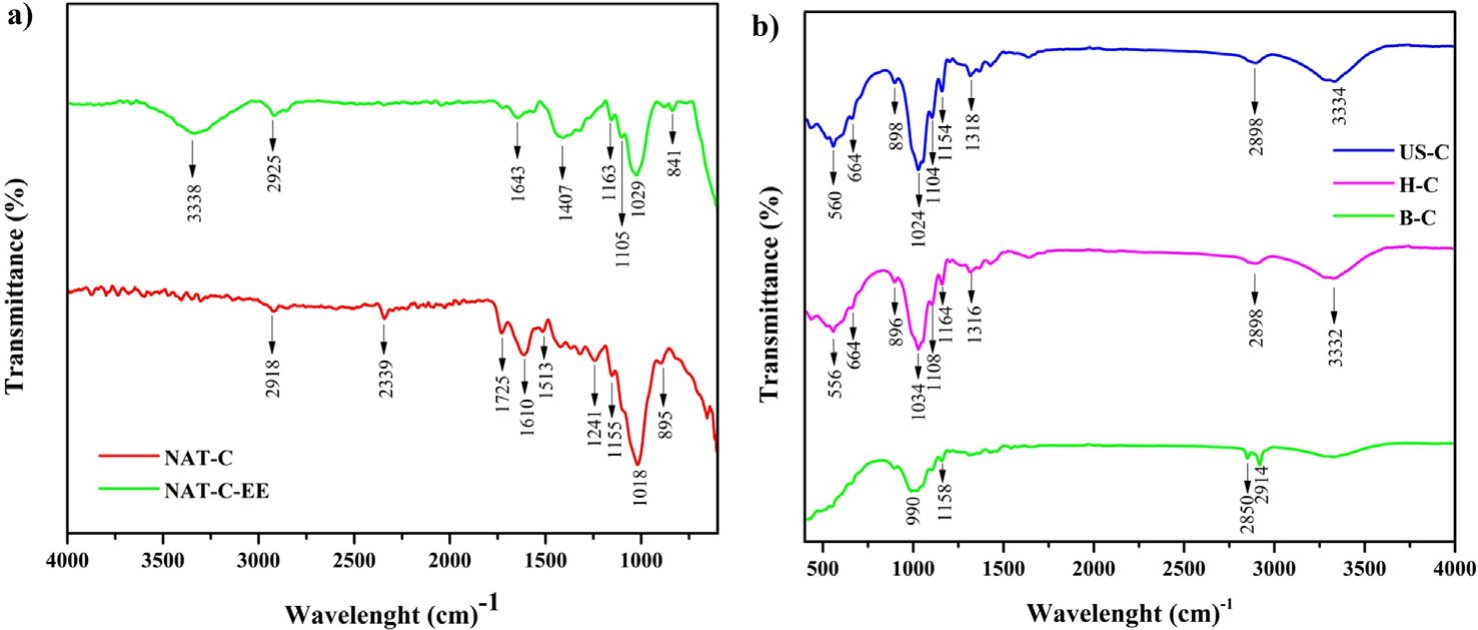
FTIR of *P. peruviana* fibers (a): untreated native fibers (NAT-C) and native fibers after the pectin and fat extraction process (NAT-C-EE). b: bleached fibers (B–C), hydrolyzed fibers (H–C), fibers treated with ultrasound (US–C).

Infrared spectrum (Figure 2 a and b) show two absorption zones, a low wavelength region (between 750 to 1750 cm^−1^) and a high wavelength region (between 2800 and 3800 cm^−1^). NAT-C presented an absorption band at 1727 cm^−1^, and it corresponds to bond vibration present in the ester and carboxylic groups (-COOCH_3_ and –COOH) characteristic of hemicellulose, ferulic, and p-coumaric acids, which belongs to lignin (Julie Chandra et al., 2016; Ovalle-Serrano et al., 2018).

This peak disappeared with the delignification after treatment with NaOH. The band at 1246 y 1612 cm^−1^ (NAT-C) corresponds to -C-O-C- bond vibration in the bonds between the aromatic ring and methoxy groups in lignin (Ovalle-Serrano et al., 2018). The FTIR of B–C, H–C, and US-C show the disappearance of several peaks (1640, 1650, 1600, and 1400 cm-^1^) caused by lignin removal hemicellulose to chemical treatment. Similar results were observed by Julie Chandra et al. (2016).

The peaks that appear at 1100 and 1000 cm^−1^ (B–C, H–C, and US-C) are attributed to the C–O–C stretching vibrations of the B-1,4, glycosidic ring bonds between the D-glucose units in cellulose (Julie Chandra et al., 2016). Also, typical cellulose signals observed in the spectra include the bending of C_6_–H_2_ between 1426 and 1323 cm^−1^ (Ovalle-Serrano et al., 2018).

The presence of characteristic cellulose signals confirms surface oxidation (Ovalle-Serrano et al., 2018). This behavior indicates that the molecular structures of cellulose remained unchanged in the presence of an acid hydrolysis process.

#### 3.1.3 Thermogravimetric analysis (TGA)

**Figure 3:**
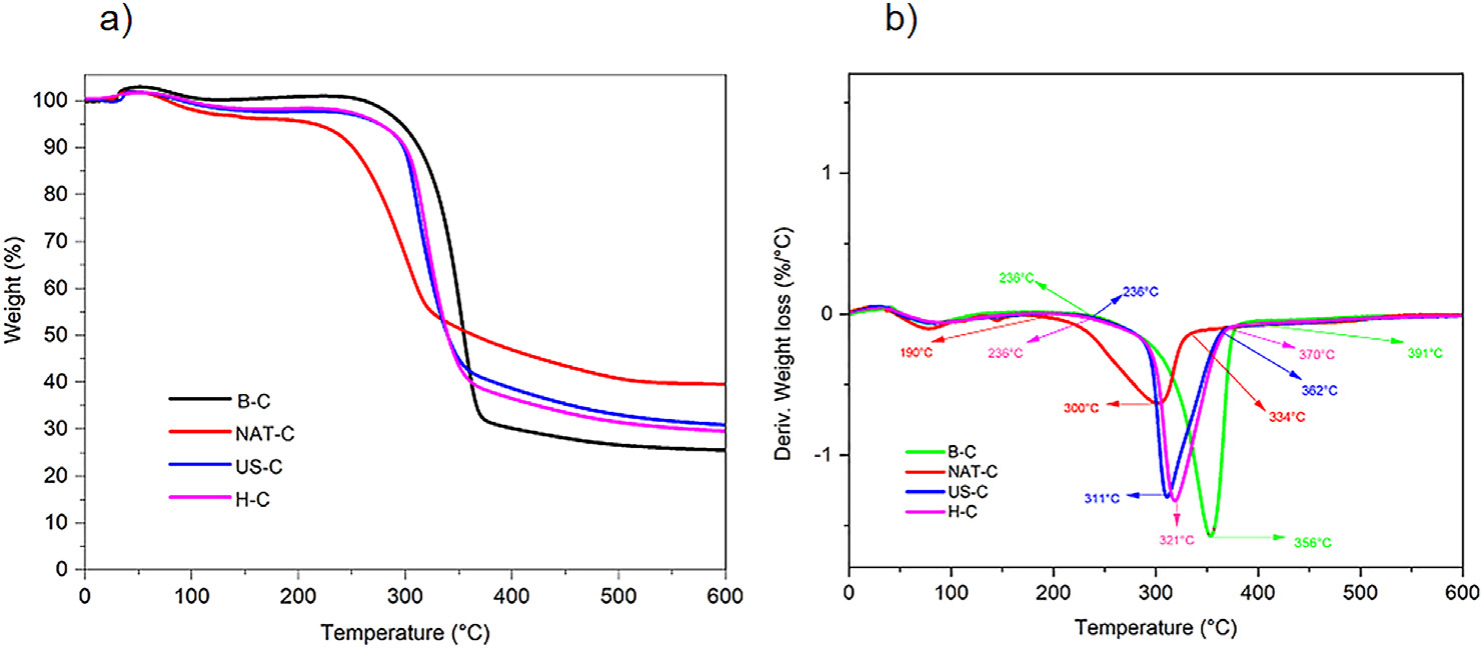
TGA analysis of *P. peruviana* fibers (a) untreated native fibers (NAT-C), bleached fibers (B–C), hydrolyzed fibers (H–C), ultrasound treated fibers (US–C). (b) Derived thermogravimetric analysis of gooseberry calyx fibers: untreated native fibers (NAT-C), bleached fibers (B–C), hydrolyzed fibers (H–C), ultrasound treated fibers (US–C).

TGA was developed to demonstrate the efficiency of bleaching, acid hydrolysis, and ultrasound treatment (Figure 3). Three degradation stages were observed: the first, below 200 °C, the second between 200 °C and 400 °C, and the third above 400 °C (Figure 3b). Weight loss due to moisture removal in the range of 30 °C–150 °C was observed (Figure 3a). Cellulosic materials showed maximum degradation in the temperature range of 300 °C–360 °C. The initial degradation temperature increased from 190 °C (NAT-C) to 236 °C (B–C, H–C, and US-C). This can be attributed to the partial removal of hemicellulose, lignin, and pectin in the delignification and bleaching process (Figure 3b). Similar results were obtained by Hoyos et al. (2019). The degradation temperature of B–C was identical to commercial cellulose (CC), while the degradation temperature of H–C and US-C was lower than the degradation temperature of CC (338 °C). The degradation temperature results were close to those reported by Ovalle-Serrano et al. (2018) in nanofibers from fique waste.

### 3.2 Physical and physicochemical analyses

#### 3.2.1 Appearance

**Figure 4:**
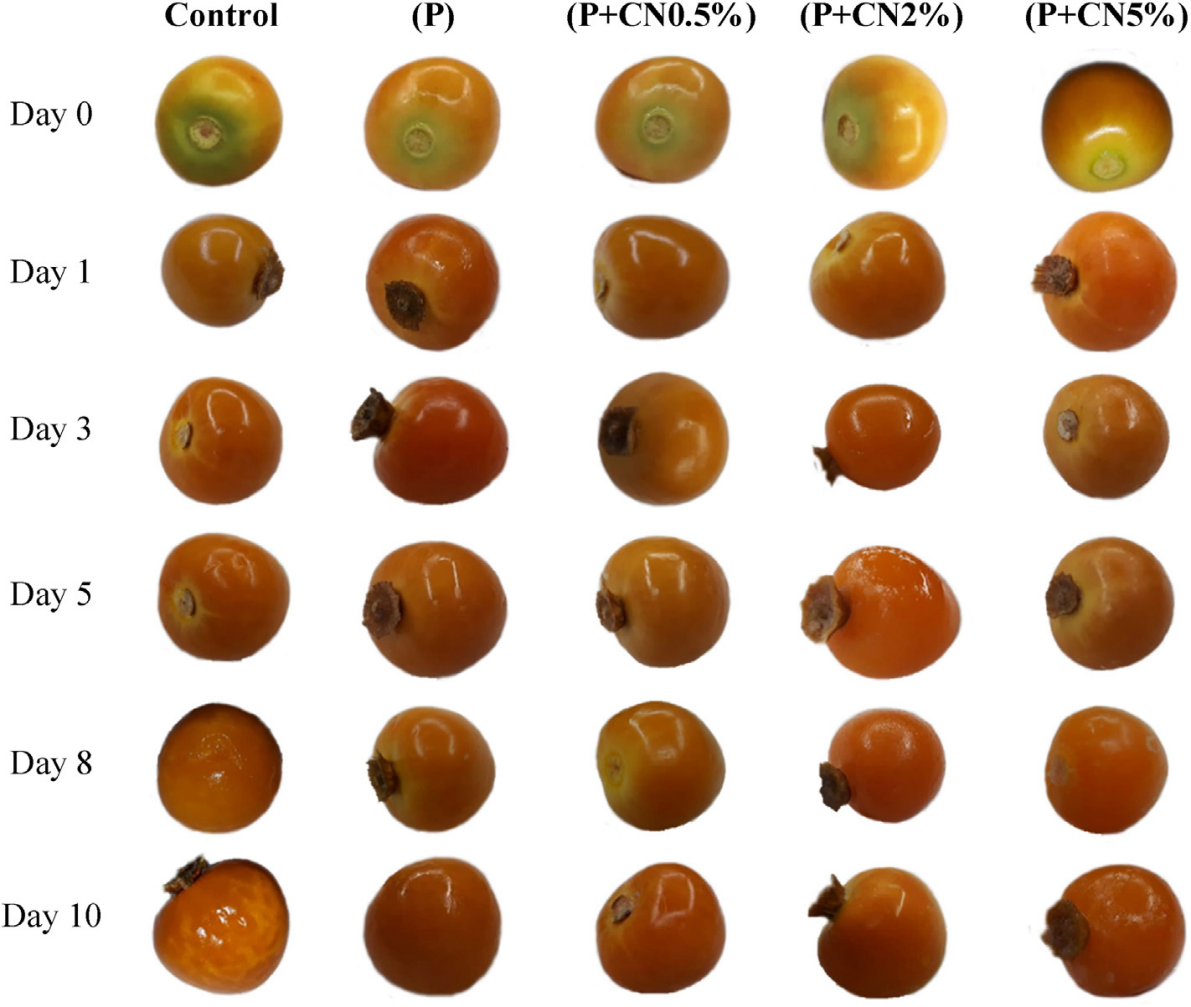
*P. peruviana* fruits coated with an edible coating based on pectin (P) and pectin (P) reinforced with different levels of cellulose nanofibers (CN) and stored for 10 days at 5 ± 2 °C and 60 ± 5% RH.

Figure 4 shows the appearance of *P. peruviana* fruits coated in comparison with the control group. The fruits showed a change from green (near the calyx area) to orange on the first day. On the 5th day, the fruits of the treatment P + CN2% presented swelling due to hydration. On the 8th day of storage, 10% of the fruits of the control group showed cracking in the epidermis in the equatorial region (the fruits that presented cracking were discarded). The fruits of the P + CN5% treatment gave swelling. The fruits of the treatment P and P + CN0.5% showed firm consistency and smooth rind during ten days of observation. The changes in the color of the fruits can be explained as a physiological response of the fruit to the stress generated by the separation of the calyx. The calyx represents a source of carbohydrates during the growth and maturity of the fruit (Bazana et al., 2019). The separation of the calyx influences the acceleration of the maturation and senescence processes (M.-L. Olivares-Tenorio et al., 2017). The cracking in the epidermis of the fruits is caused by the increase in internal pressure during the ripening processes (Fischer et al., 2014). Cracking is the biggest defect in the visual quality of *P. peruviana* fruits (Mayorga-Cubillos et al., 2019).

Nanocellulose has previously been used to reduce cherry cracking (Jung et al., 2016). The swelling of the fruits treated with pectin reinforced with the highest concentration of nanocellulose is attributed to the hydrophilic nature of the films based on polysaccharides (Dai et al., 2016). This indicates that coatings reinforced with nanocellulose are hygroscopic and can absorb moisture released in the perspiration of the fruit and the environment. Therefore, higher concentrations of nanocellulose generate slower diffusion processes. Pacaphol et al. (2019) mentioned that the hydroxyl groups (-OH) present on the surface of nanocellulose could establish strong hydrogen bonds to hold the H_2_O molecules.

On the other hand, the treatment with a lower amount of nanocellulose (P + NC0.5%) did not show hydration of the coating. Therefore, a low concentration of the polymer generates a greater regulation in the migration of H_2_O molecules between the fruit and the environment. Dai et al. (2016) evaluated the addition of hydroxypropyl guar and carboxymethyl guar to nanocellulose composite films. The authors mentioned that the presence of –OH groups and the pore size in the film are factors that affect water absorption. Similar results were reported by Pacaphol et al. (2019) when applying suspensions of nanocellulose 0.5% in spinach.

#### 3.2.2 Weight loss

**Figure 5:**
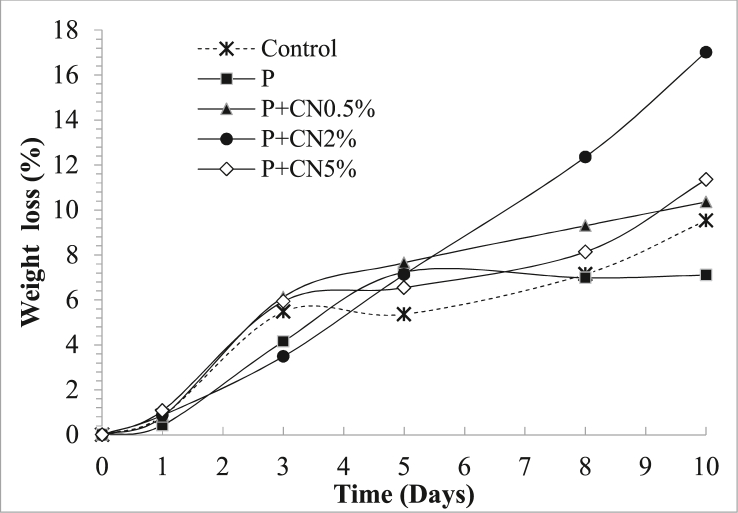
Effect of edible coatings of pectin (P) and pectin (P) reinforced with cellulose nanofibers (coating reinforced at 0.5% (P + CN0.5%), coating reinforced at 2% (P + CN2%) and coating reinforced at 5% (P + CN5%)) on weight loss the *P. peruviana* fruits during storage at 5 ± 2 °C and 60 ± 5% RH. Error bar shows standard deviation.

Weight loss of control and treated samples all along the storage are presented in Figure 5. In all treatments, there are no significant differences until day 5 of storage. However, on days 8 and 10, the P + CN2% treatment showed a significant difference with respect to the other treatments. The lowest weight loss occurred in fruits coated with pectin (7.10%), followed by the control treatment. The greatest weight loss was obtained with the P + CN2% treatment (17.01%), followed by P + CN5%.

A progressive weight loss occurred in the fruits coated with nanocellulose. This behavior can be attributed to the respiration of fruits (Silva-Vera et al., 2018). The greater weight loss in the fruits coated with reinforced pectin is attributed to the swelling of the coating due to the adsorption of water (hydrophilic polymer). This phenomenon increases the diffusion of H_2_O molecules from the fruit to the environment (Md Nor and Ding, 2020). Although, the addition of nanocellulose to the pectin coating did not favor the reduction in weight loss. Other authors have reported that nanocellulose reduces the weight loss of Tahitian lime (Laureth et al., 2018) and slices of apples (Zhai et al., 2020). The greater reduction in weight loss of the pectin (P) coated fruits is attributed to a greater ability to decrease the transport of water molecules. This capacity is attributed to the plasticization generated with glycerin by the presence of hydrophobic groups (Guerreiro et al., 2016). This phenomenon reduces perspiration and maintains turgor pressure on the cell walls of the fruit (Laureth et al., 2018). The present study results are consistent with what was reported by Estrada-Girón et al. (2020).

#### 3.2.3 Fruit firmness

**Figure 6:**
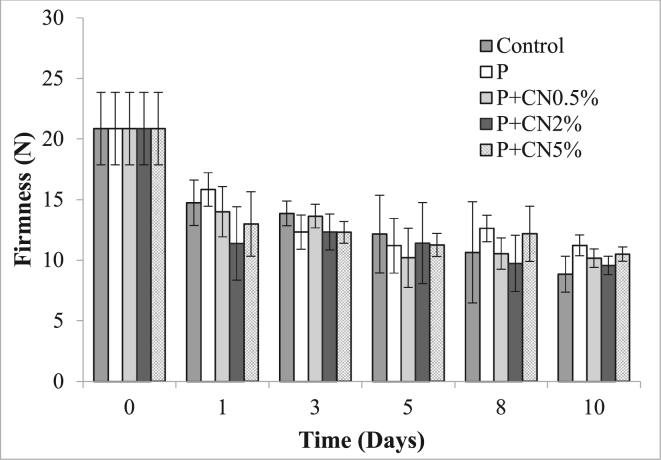
Firmness of *P. peruviana* fruits subjected to different treatments during storage at 5 ± 2 °C and 60 ± 5% RH: untreated (control), only pectin (P), pectin coating reinforced with cellulose nanofibers at 0.5% (P + CN0.5%), pectin-coating reinforced with cellulose nanofibers at 2% (P + CN2%) and pectin-coating reinforced with cellulose nanofibers at 5% (P + CN5%). Error bar shows standard deviation.

Figure 6 shows the changes in the firmness of *P. peruviana* fruits subjected to different treatments during storage at 5 ± 2 °C and 60 ± 5% RH. Firmness values decreased throughout storage for all treatments, possibly due to the senescence process. On day 10, the control treatment presented the lowest firmness value (8.84 ± 1.4 N). However, statistically significant differences between the treatments were not observed.

Firmness and water loss are parameters that are related. The loss of firmness may be associated with an increase in the respiratory rate in fruits, this is related to the ripening of the fruit, pectin methylesterase, and polygalacturonase, hydrolyze pectin causing changes in its solubility in the cell wall, this causes a gradual softening in the epidermis of *P. peruviana* (M.-L. Olivares-Tenorio et al., 2017). The fruits coated with pectin (P) presented the least weight loss and showed the highest firmness retention at the end of storage. Similar results were reported by Sarduni et al. (2020) in coatings of pectin (1, 2, and 3%, w/v) and sodium chloride applied to Berangan bananas stored for 5 days at 25 ± 2 °C.

Deng et al., 2017a, Deng et al., 2017b found that applying a coating of oleic acid and sucrose ester fatty acid based on nanocellulose was able to preserve firmness in Cavendish bananas stored for 10 d. The application of a nanocomposite made of pectin and nanocellulose can retain the firmness in the fruits of Tahiti acid lime (stored for 9 days at 23 ± 2 °C and 75% RH) compared to formulations without nanocellulose (Laureth et al., 2018).

#### 3.2.4 Determination of color

**Figure 7:**
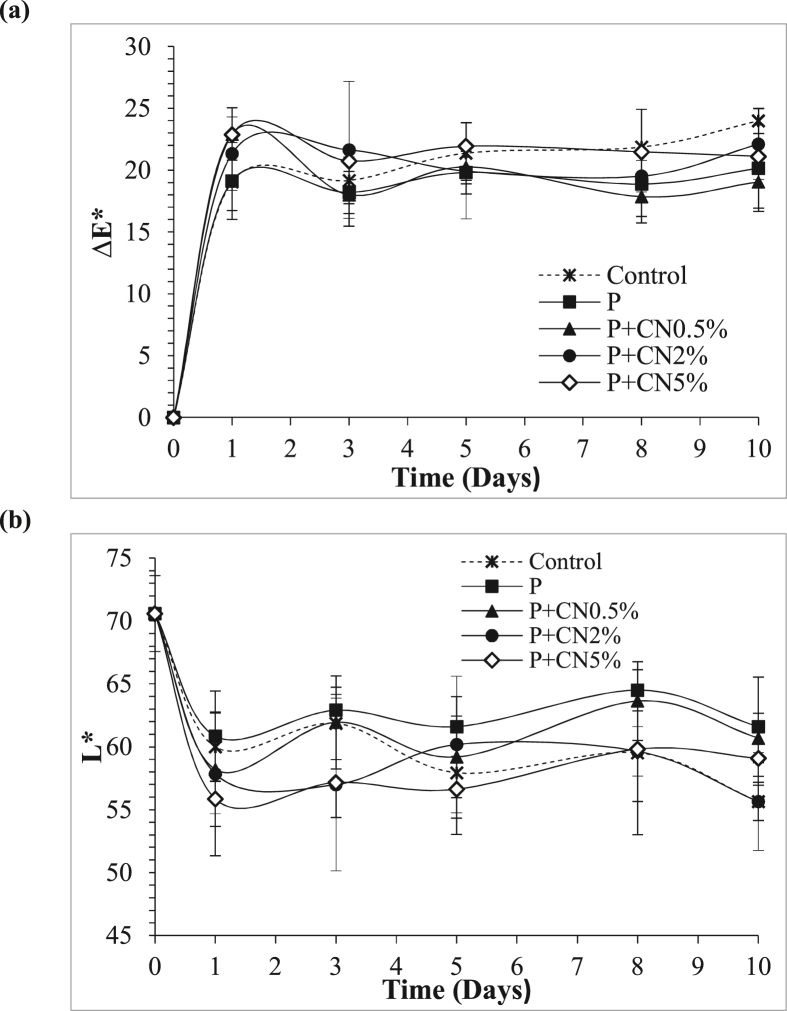
Total color change ΔE (a) and luminosity L∗(b) of *P. peruviana* fruits subjected to different treatments during storage at 5 ± 2 °C and 60 ± 5% RH: untreated (control), only pectin (P), pectin-coating reinforced with cellulose nanofibers at 0.5% (P + CN0.5%), pectin-coating reinforced with cellulose nanofibers at 2% (P + CN2%) and pectin-coating reinforced with cellulose nanofibers at 5% (P + CN5%).

The total changes in the color of the epidermis of *P. peruviana* fruits are shown in Figure 7a. The type of coating did not present a statistical significance for ΔE. The lowest color change at the end of storage was observed in the treatments P + CN0.5% (19.04) and P (21.11) compared to the control (23.98). Figure 7b shows the change in the L∗ luminosity values of the *P. peruviana* fruits for all the treatments. On day 3 of storage, P, P + CN2%, and P + CN5% showed a significant difference. On day 10, the difference was between the control, P, and P + CN2% treatments. The Coatings P and P + CN0.5% presented the highest values of L∗.

Color changes in *P. peruviana* fruits are generated by the degradation of chlorophyll and the synthesis of pigments such as carotenoids (Wen et al., 2020). During the first day, the increase in color change can be affected by the increase in the respiratory rate due to the separation of the calyx. Therefore, the lower color variation obtained with the P + CN 0.5% treatment may be related to a decrease in the respiratory rate. These results are consistent with those reported by Deng et al., 2017a, Deng et al., 2017b, in chitosan coatings (2%) reinforced with cellulose nanocrystals (0, 5, and 10% w/w) applied to pears stored for 3 weeks at 20 ± 2 °C and 30 ± 2% RH. The authors found that the treated fruits retained the green pigment and presented lower ΔE values than the control of fruits.

Treatments P and P + CN0.5% showed the highest retention of L∗. These results coincide with the best-looking treatments (firm consistency with shiny surfaces). The fruits treated with coatings reinforced with the highest concentration of nanocellulose showed opacity in the epidermis; this behavior could be associated with dehydration of the surface (Ebrahimi and Rastegar, 2020). These results are consistent with the reported by Sarduni et al. (2020), in Berangan bananas coated with pectin (1, 2, and 3 %, w/v) and sodium chloride, stored for 5 days at 25 ± 2 °C. Pacaphol et al. (2019), reported that the 0.5% nanocellulose suspension retains the green color and luminosity of spinach leaves stored for 3 days (in accelerated conditions of 25 ± 0.5 °C and 40–56% RH).

#### 3.2.5 Potential of hydrogen (pH), total soluble solids (TSS), titratable acidity (TA), and (TSS/TA) ratio

**Figure 8:**
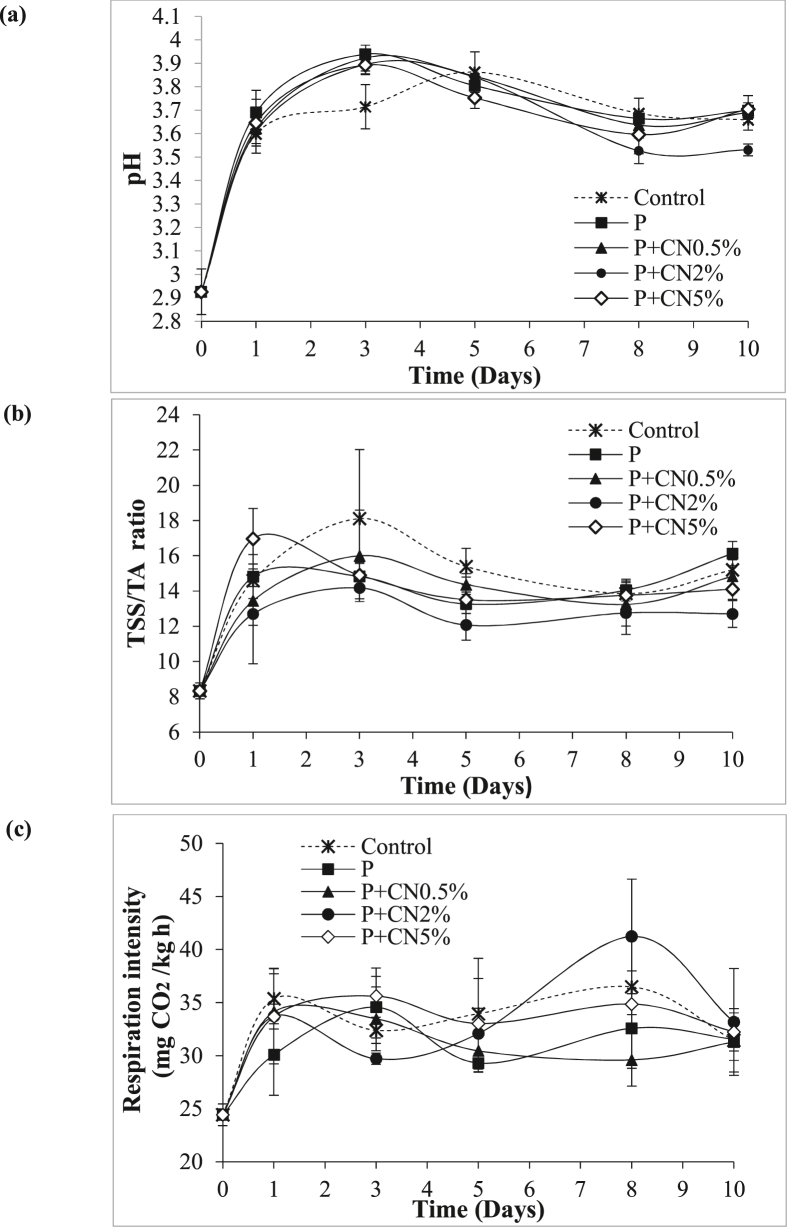
Effect of edible coatings of pectin (P) and pectin reinforced with cellulose nanofibers (coating reinforced at 0.5% (P + CN0.5%), coating reinforced at 2% (P + CN2%) and coating reinforced at 5% (P + CN5%)) on pH (a), SS/TA ratio (b) and respiration rate (c) of *P. peruviana* fruits during storage at 5 ± 2 °C and 60 ± 5% RH. Error bar shows standard deviation.

The pH of *P. peruviana* fruits showed a variation in storage time (Figure 8a). The control, P, and P + CN2% treatments presented significant differences. The pH values progressively increased until day 3 in the treatments. In control, the increase was until day 5. On day 10, significant differences were presented between P + CN2% and the other treatments. This treatment had the lowest pH values (3.53 ± 0.02), the P + CN5% treatment showed the highest pH value (3.70 ± 0.06). The increase in pH values during the first days of storage is associated with accelerating the ripening processes that the fruit undergoes due to the consumption of organic acids. Zhai et al. (2020) reported that the pH values are used to indicate the state of maturity. These results are comparable with those obtained by Silva-Vera et al. (2018) in grapes coated with nanocellulose (slight increase in pH values).

The P + CN2% treatment showed a significant effect on the values of TSS/TA (Figure 8b), TA, and TSS (Table 1) with respect to the other treatments. In general, all treatments showed a peak in TSS/TA values between days 1 and 3. Treatments P and P + CN5% presented the peak on the first day of storage; the other treatments presented the peak on day 3. This peak is attributed to the adaptation of the fruit to the storage conditions. The control treatment presented significantly the highest TSS/TA values after the second day of storage. After day 3, the fruits experienced a decrease in the TSS/TA values. These results show a reduction in the maturation processes of the coated fruits. This is attributed to the regulation of metabolic activities, consumption of organic acids, and the conversion of polysaccharides into simple sugars. These results may be influenced by the low permeability presented by pectin and nanocellulose. These results are consistent with the study of López-Enríquez et al. (2016). The authors demonstrated that the application of a coating of beeswax and whey proteins decreases the TSS/TA values in *P. peruviana* fruits stored for 15 days at 17 ± 2 °C and RH 69%. Likewise, the application of nanocellulose delayed the ripening process of grapes stored at 4 ± 0.5 °C for 41 days compared to uncoated fruits (Silva-Vera et al., 2018).

**Table 1:** TSS and TA values of *P. peruviana* fruits coated with pectin (P) or pectin reinforced with cellulose nanofibers (P + CN) during storage at 5 ± 2 °C and 60 ± 5%. P = pectin; P + CN0.5% = coating reinforced at 0.5%; P + CN2% = coating reinforced at 2%; P + CN5% = coating reinforced at 5%.

Treatment	Time (Days)					
	0	1 d	3 d	5 d	8 d	10 d
TSS						
Control	13.80 ± 0.21^a^	14.20 ± 0.37^a^	14.86 ± 0.27^c^	15.72 ± 0.46^c^	15.14 ± 0.61^b^	15.70 ± 0.10^c^
P	13.80 ± 0.21^a^	14.21 ± 0.48^a^	13.90 ± 0.29^ab^	14.14 ± 0.32^b^	13.82 ± 0.53^a^	15.46 ± 0.43^bc^
P + CN0.5%	13.80 ± 0.21^a^	14.14 ± 0.49^a^	14.26 ± 0.58^ab^	14.48 ± 0.63^b^	14.52 ± 0.67^ab^	15.72 ± 0.46^c^
P + CN2%	13.80 ± 0.21^a^	13.77 ± 0.40^a^	13.74 ± 0.60^a^	13.86 ± 0.49^b^	14.90 ± 0.80^b^	14.62 ± 0.56^a^
P + CN5%	13.80 ± 0.21^a^	13.96 ± 0.36^a^	14.36 ± 0.24^bc^	12.96 ± 0.29^a^	14.84 ± 0.35^b^	15.00 ± 0.23^ab^
TA (% citric acid)						
Control	1.66 ± 0.10^a^	0.97 ± 0.01 ^ab^	0.85 ± 0.19^a^	1.02 ± 0.06 ^ab^	1.10 ± 0.12 ^ab^	1.02 ± 0.04^b^
P	1.66 ± 0.10^a^	0.96 ± 0.08 ^ab^	0.94 ± 0.05^a^	1.07 ± 0.07 ^bc^	0.99 ± 0.09^a^	0.97 ± 0.06^a^
P + CN0.5%	1.66 ± 0.10^a^	1.06 ± 0.11^b^	0.91 ± 0.14^a^	1.06 ± 0.05 ^ab^	1.11 ± 0.19 ^ab^	1.08 ± 0.05^b^
P + CN2%	1.66 ± 0.10^a^	1.12 ± 0.30^b^	0.97 ± 0.05^a^	1.15 ± 0.11^c^	1.17 ± 0.09^b^	1.15 ± 0.07^c^
P + CN5%	1.66 ± 0.10^a^	0.82 ± 0.12^a^	0.97 ± 0.04^a^	0.97 ± 0.14^a^	1.08 ± 0.08 ^ab^	1.08 ± 0.06^b^

The values are mean ± SD (n = 5), the means with different superscript in a column are significantly different (P < 0.05).

In all the treatments, the TA of the fruits decreased at the beginning of storage (Table 1). This is attributed to the use of organic acids as substrates in respiratory processes (Zhang et al., 2019). At the end of storage, the fruits coated with the P + CN2% treatment showed high TA values (P < 0.05). This indicates that nanocellulose reduces the consumption of organic acids as a primary source in the respiratory processes of the fruit. These results agree with those obtained from freshly cut apple slices coated with nanocellulose (2% w/w) and stored at 4 °C for 7 days (Zhai et al., 2020). These authors indicated that nanocellulose suspensions could decrease the respiratory rate of fruits. Similar results were also reported in applying a nanocellulose-based coating on Cavendish bananas stored for 10 days (Deng et al., 2017a, Deng et al., 2017b). The decrease in the acidity of treatment P is attributed to the barrier properties obtained with this treatment. The low concentration of the hydrophilic polymer in the formulation can induce anaerobiosis (increased consumption of organic acids). This behavior was mentioned by Deng, Jung, Simonsen, Wang, et al. (2017a) in chitosan (2%) and nanocellulose (0, 5, and 10% w/w) coatings applied to pears stored at 20 ± 2 °C for 3 weeks. The authors found that the fruits coated with chitosan presented lower acidity values compared to the control fruits. This behavior was attributed to the fact that the fruits used organic acids as an energy reserve under the anaerobic conditions of the coating.

*P. peruviana* fruits showed a gradual increase in TSS content during storage; this is explained by the hydrolysis of polysaccharides (converted into disaccharides and monosaccharides) as a result of the increase in enzymatic activity (Ebrahimi and Rastegar, 2020). In this investigation, *P. peruviana* coated fruits kept low TSS concentrations compared to the control group. This result is attributed to the decrease in the respiratory rate (reduction of the metabolic process) presented in the coated fruits. The application of pectin and nanocellulose polymers as a coating with a high barrier to gases that intervene in respiration can delay the ripening processes of the fruit. This result is in accordance with that reported by López-Enríquez et al. (2016) *P. peruviana* fruits coated with beeswax and whey proteins. Similar results were also reported by Sarduni et al. (2020) and Deng et al., 2017a, Deng et al., 2017b in bananas coated with pectin (1, 2, and 3%, w/v) and nanocellulose (0, 5, and 10% w/w), respectively.

### 3.3 Evaluation of the respiration rate of the fruits

Figure 8c shows the respiration rate of coated *P. peruviana* fruits and control. Although the treatments did not show a significant difference, fruits coated with P + CN0.5% presented the lowest respiratory rate throughout the experiment (29.60 mgCO_2_/kg h). This treatment showed a respiratory peak on day 1. The treatments maintained the tendency to have two peaks on days 1 and 8 (except for P). The evaluation of the respiratory rate showed that *P. peruviana* fruits have climacteric respiration (due to the marked peaks of carbon dioxide production). The climacteric behavior patterns in *P. peruviana* fruits have been previously reported by Junqueira et al. (2017) and López-Enríquez et al. (2016). The treated fruits showed two peaks in respiration; the presence of the pre-climacteric peak can be attributed to the response to stress generated by the separation of the calyx and adaptation to the conditions of the experiment. The Determination of the second peak (climacteric) on day 8 is in accordance with that reported by López-Enríquez et al. (2016) in *P. peruviana* fruits coated with whey proteins and beeswax. The lower respiratory rate determined in the P + CN0.5% treatment can be attributed to a lower amount of the hydrophilic compound. This coating can delay the ripening processes by acting as a low diffusion barrier to gases. This property is attributed to forming hydrogen bonds and the high crystalline content of nanocellulose (Ventura-Cruz and Tecante, 2019). Similar results were reported by Pacaphol et al. (2019) in spinach leaves coated with 0.5% nanocellulose stored for 3 days at 25 ± 0.5 °C and 40–56% RH. The results of the present study are also consistent with those obtained by Laureth et al. (2018) in Tahiti acid lime coated with pectin and nanocellulose. The authors reported that the coating of pectin and nanocellulose could form a more efficient gas barrier than the application of pure pectin. The good results obtained in this study open the possibility in future studies to optimize the parameters of coatings that extend the shelf life of *P. peruviana.*

## 4 Conclusions

Nanocellulose was successfully extracted and characterized from *P. peruviana* calyces. The application of pectin or pectin reinforced with nanocellulose at 0.5% (w/w) has a positive effect on some physicochemical properties of *P. peruviana*; the fruits showed an adequate visual appearance, minor changes in color index, and less respiratory intensity during 10 days of storage compared to the other treatments and the control. However, the application of pectin alone showed less weight loss and greater firmness. Therefore, edible pectin-coating could be used to prolong the shelf life of *P. peruviana* fruits. Edible pectin coatings reinforced with nanocellulose from *P. peruviana* calyces can be used under the concept of the circular economy. However, this application has limitations with respect to the use of pectin alone.

## Declarations

### Author contribution statement

Liceth Carolina Cárdenas-Barboza: Conceived and designed the experiments; Performed the experiments; Analyzed and interpreted the data; Wrote the paper.

Andrey Camilo Paredes-Córdoba, Marcelo Guancha-Chalapud: Performed the experiments; Contributed reagents, materials, analysis tools or data.

Liliana Serna-Cock: Conceived and designed the experiments; Contributed reagents, materials, analysis tools or data; Wrote the paper.

Cristian Torres-León: Analyzed and interpreted the data; Wrote the paper.

### Funding statement

This work was supported by the National University of Colombia (UNAL, Palmira, Colombia) and by the National Center for Technical Assistance to Industry (ASTIN, Cali, Colombia).

### Data availability statement

Data will be made available on request.

### Declaration of interests statement

The authors declare no conflict of interest.

### Additional information

No additional information is available for this paper.
